# Combination of cilostazol and clopidogrel attenuates Rat critical limb ischemia

**DOI:** 10.1186/1479-5876-10-164

**Published:** 2012-08-16

**Authors:** Jiunn-Jye Sheu, Kun-Chen Lin, Ching-Yen Tsai, Tzu-Hsien Tsai, Steve Leu, Chia-Hung Yen, Yung-Lung Chen, Hsueh-Wen Chang, Cheuk-Kwan Sun, Sarah Chua, Jenq-Lin Yang, Hon-Kan Yip

**Affiliations:** 1 Division of Thoracic and Cardiovascular Surgery, Chang Gung Memorial Hospital- Kaohsiung Medical Center, Chang Gung University College of Medicine, Gueishan, Taiwan; 2 Department of Anesthesia Chang Gung Memorial Hospital-Kaohsiung Medical Center, Chang Gung University College of Medicine, Gueishan, Taiwan; 3 Institute of Molecular Biology, Academia Sinica, Taipei, 115, Taiwan; 4 Division of Cardiology, Department of Internal Medicine; Chang Gung Memorial Hospital- Kaohsiung Medical Center, Chang Gung University College of Medicine, Gueishan, Taiwan; 5 Center for translational research in biomedical science, Chang Gung Memorial Hospital- Kaohsiung Medical Center, Gueishan, Taiwan; 6 Department of Life Science, National Pingtung University of Science and Technology, Neipu, Taiwan; 7 Department of Biological Sciences, National Sun Yat-Sen University Kaohsiung, Kaohsiung, Taiwan; 8 Department of Emergency Medicine, E-Da Hospital, I-Shou University, Kaohsiung, Taiwan; 9 Division of Cardiology, Department of Internal Medicine, Chang Gung Memorial Hospital, Kaohsiung 123, Ta Pei Road, Niao Sung Dist, Kaohsiung City, 83301, Taiwan

**Keywords:** Critical limb ischemia, Apoptosis, Inflammation, Pharmacomodulation, Angiogenesis

## Abstract

**Background and aim:**

Procedural failure and untoward clinical outcomes after surgery remain problematic in critical limb ischemia (CLI) patients. This study tested a clopidogrel-cilostazol combination treatment compared with either treatment alone in attenuating CLI and improving CLI-region blood flow in rats.

**Methods:**

Male Sprague–Dawley rats (n = 40) were equally divided into five groups: control, CLI induction only, CL I + cilostazol (12.0 mg/day/kg), CLI + clopidogrel (8.0 mg/kg/day) and CLI + combined cilostazol-clopidogrel. After treatment for 21 days, Laser Doppler imaging was performed.

**Results:**

On day 21, the untreated CLI group had the lowest ratio of ischemic/normal blood flow (p < 0.001). Inflammation measured by VCAM-1 protein expression; oxidative stress; PAI-1, MMP-9 and TNF-α mRNA expressions; and immunofluorescence staining (IF) of CD68+ cells was lower with combined treatment than with the other treatments, and lower in the two single-treatment groups than the untreated CLI group (all p < 0.01). Anti-inflammatory mRNA expression of interleukin-10, and eNOS showed a reverse pattern among these groups. Apoptosis measured by Bax, caspase-3 and PARP; and muscle damage measured by cytosolic cytochrome-C, and serum and muscle micro-RNA-206 were all lowest with combination treatment, and the two single-treatment groups showed lower values than the untreated group (all p < 0.001). Angiogenesis measured by eNOS, IF staining of CD31+ and vWF + cells; and number of vessels in CLI region were highest with combination treatment and higher in the single-treatment groups than the untreated group (all p < 0.001).

**Conclusion:**

Combined cilostazol-clopidogrel therapy is superior to either agent alone in improving ischemia in rodent CLI.

## Background

Some of the most important risk factors for atherosclerosis are hypercholesterolemia, hypertension, and diabetes mellitus. Peripheral arterial disease (PAD) is characterized by atherosclerotic occlusion of the lower extremities and is a common manifestation of systemic atherosclerosis [1]. Patients with PAD have approximately the same relative risk of death from cardiovascular causes as those with a history of coronary or cerebrovascular disease [2], and patients with PAD may develop critical limb ischemia (CLI) at later stages of the disease [3,4].

Treatment of CLI remains a formidable challenge to the clinician. Without appropriate treatment, the 1-year mortality rate can be as high as 25% [5]. Bypass surgical intervention is one of the most popular and standard methods with the highest success rate, but procedural failure and short-term and long-term untoward clinical outcomes in some patients remain problematic. Failure to salvage CLI almost always leads to major limb loss which results in a major socio-economic burden. Therefore, development of more cost-effective treatments for patients with CLI and those unsuitable for either surgical or percutaneous intervention is urgently needed.

Clopidogrel, an adenosine diphosphatase (ADP) inhibitor, is currently used in acute arterial occlusive syndrome for inhibiting platelet activity and thrombus formation [6-9]. Additionally, cilostazol, a phosphodiesterase III inhibitor approved by the US Food and Drug Administration (FDA) for treatment of intermittent claudication, has been shown to have pleiotropic effects, including reducing smooth muscle proliferation, limiting intimal hyperplasia after endothelial injury, lowering restenotic rate of endovascular intervention, and inhibiting platelet activation and thrombus formation, as well as reducing inflammation [10-12]. Numerous clinical trials have shown that use of dual antiplatelet agents such as combined clopidogrel and aspirin are more effective than either one used alone for reducing future major adverse cardiac events in patients with acute coronary syndrome undergoing percutaneous coronary intervention [13-15]. However, whether combined cilostazol and clopidogrel therapy can offer similar additional benefit to improve PAD is currently unclear. This study used a rodent CLI model to explore the effects of a combined regimen of cilostazol and clopidogrel on ischemia in rodent CLI.

## Materials and methods

### Ethics

All animal experimental procedures were approved by the Institute of Animal Care and Use Committee at Kaohsiung Chang Gung Memorial Hospital and performed in accordance with the Guide for the Care and Use of Laboratory Animals (NIH publication No. 85–23, National Academy Press, Washington, DC, USA, revised 1996).

### Rationale for the dosage of combined cilostazol and clopidogrel

First, the safety and efficacy of different combinations of cilostazol and clopidogrel were compared. Six animals were used. They were assigned to receive low [cilostazol (8.0 mg/kg/day) and clopidogrel (4.0 mg/kg/day)], moderate [cilostazol (12.0 mg/kg/day) and clopidogrel (8.0 mg/kg/day)] or high [cilostazol (16.0 mg/kg/day) and clopidogrel (12.0 mg/kg/day)] dose regimens of this combined therapy. The high-dose regimen of this combined therapy easily induced wound swelling and bleeding after the CLI procedure. On the other hand, wound bleeding infrequently occurred in animals that received the moderate and low regimens of combined therapy. The blood flow in the CLI was markedly increased at the moderate dosage in comparison with that with the low dosage of this combined therapy. Therefore, the moderate dosage of the combined therapy, cilostazol (12.0 mg/kg/day) and clopidogrel (8.0 mg/kg/day), was used in subsequent experiments.

### Treatments

Male Sprague–Dawley rats (n = 40) were equally divided into five groups: group 1 (control), group 2 (CLI only), group 3 [CLI + cilostazol (12.0 mg/day/kg)], group 4 [CLI + clopidogrel (8.0 mg/kg/day)], and group 5 (combined cilostazol-clopidogrel) after CLI induction. All treatments were initiated just after the CLI procedure and continued for 21 days. The rats were sacrificed on day 21 after the Laser Doppler was performed.

### Flow cytometric quantification of endothelial progenitor cells

**Figure 1:**
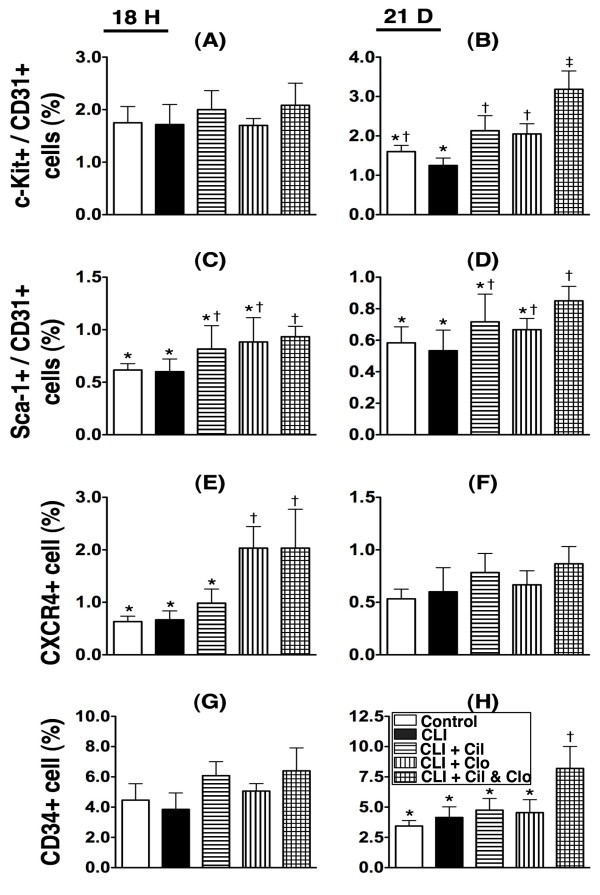
**Flow cytometric quantifications of circulating numbers of endothelial progenitor cells (EPCs) at 18 h and day 21 after critical limb ischemia (CLI) procedure (n = 8). A** &**B**) circulating number of C-kit/CD31+ cells at 18 h and day 21. By day 21,* vs. other groups with different symbols (i.e.,* vs. † vs. ‡), p < 0.001. **C** &**D**) circulating number of Sca-1/CD31+ cells at 18 h and day 21. At 18 h,* vs. other groups with different symbols, p < 0.008. At day 21,* vs. other groups with different symbols, p < 0.01. **E** &**F**) circulating number of CXCR4+ cells at 18 h and day 21. At 18 h,* vs. other groups with different symbols, p < 0.001. **G** &**H**) circulating number of CD31+ cells. By day 21,* vs. other groups with different symbols, p < 0.001. Statistical analysis in each group by ANOVA followed by Bonferroni multiple comparison post hoc test. CLI = critical limb ischemia; Cil = cilostazol; Clo = clopidogrel.

For blood sampling at different time points (at 18 h and on day 21 after induction of CLI), the tail venous route was adopted for blood sampling using 27# needle. After treatment with red blood cell-lysing buffer for the blood sample, the cells remained were labeled with appropriate antibodies. Flow cytometric analysis for identification of cell surface markers was performed based on our recent reports [16]. Briefly, the cells were immunostained for 30 minutes with monoclonal antibodies against primary antibodies, including CD 31 (serotec), C-kit-FITC (BD biosciences), Sca-1-PE (R&D), CXCR4 (Abcam), and CD34-PE (BD biosciences). Secondary detection was performed using appropriate Alexa Fluor 488 (Molecular Probes, Eugene, OR). Isotype-identical antibodies (IgG) served as controls. Flow cytometric analyses were performed by utilizing a fluorescence-activated cell sorter (Beckman Coulter FC500 flow cytometer). **Measurement of Blood Flow with Laser Doppler (**Figure 1**)**

Rats were anesthetized by intraperitoneal injections of isoflurane (2.0%) prior to CLI induction and at days 2 and 21 after CLI induction prior to be sacrificed. The rats were placed in a supine position on a warming pad at 37 °C and the blood flow were detected in both inguinal areas by a Laser Doppler scanner (moorLDLS, Moor, Co. UK). The ratio of flow in left (ischemic) leg and right (normal) leg was computed. The rats were sacrificed and the quadriceps muscle was collected for individual study.

### Western blot analyses

Equal amounts (50 μg) of protein extracts were loaded and separated by SDS-PAGE using acrylamide gradients. After electrophoresis, the separated proteins were transferred electrophoretically to a polyvinylidene difluoride (PVDF) membrane (Amersham Biosciences). Nonspecific sites were blocked by incubation of the membrane in blocking buffer [5% nonfat dry milk in T-TBS (TBS containing 0.05% Tween 20) overnight. The membranes were incubated with the indicated primary antibodies [vascular cell adhesion molecule (VCAM)-1 (1:100, Abcam), cytochrome c (Cyt c) (1: 2000, BD), Bax (1: 1000, Abcam), caspase 3 (1:1000, Cell Signaling), poly(ADP-ribose) polymerase (PARP) (1:1000, Cell Signaling), endothelial nitric oxide synthase (eNOS) (1: 1000, Abcam), Actin (1: 10000, Chemicon )] for 1 hour at room temperature. Horseradish peroxidase -conjugated anti-rabbit immunoglobulin IgG (1: 2000, Cell Signaling) was used as a second antibody for one hour at room temperature. The washing procedure was repeated eight times within one hour, and immunoreactive bands were visualized by enhanced chemiluminescence (ECL; Amersham Biosciences) and exposure to Biomax L film (Kodak). For purposes of quantification, ECL signals were digitized using Labwork software (UVP).

#### Oxidative stress reaction of LV myocardium

The Oxyblot Oxidized Protein Detection Kit was purchased from Chemicon (S7150). DNPH derivatization was carried out on 6 μg of protein for 15 minutes according to manufacturer’s instructions. One-dimensional electrophoresis was carried out on 12% SDS/polyacrylamide gel after DNPH derivatization. Proteins were transferred to nitrocellulose membranes which were then incubated in the primary antibody solution (anti-DNP 1: 150) for 2 hrs, followed by incubation with second antibody solution (1:300) for 1 hr at room temperature. The washing procedure was repeated eight times within 40 minutes. Immunoreactive bands were visualized by enhanced chemiluminescence (ECL; Amersham Biosciences) which was then exposed to Biomax L film (Kodak). For quantification, ECL signals were digitized using Labwork software (UVP). For oxyblot protein analysis, a standard control was loaded on each gel.

#### Isolation of mitochondria from quadriceps

The skeletal muscle from ischemic region was excised and washed with buffer A (100 mM Tris–HCl, 70 mM sucrose, 10 mM EDTA and 210 mM mannitol, pH 7.4). The samples were first minced finely in cold buffer A and then incubated for 10 minutes at 4°C. All samples were then homogenized in an additional 3 mL of buffer A using a motor-driven grinder. The homogenate was centrifuged twice at 700 g for 10 minutes at 4°C. The supernatant was again centrifuged at 8,500 g for 15 minutes, and the pellets were then washed with buffer B (10 mM Tris–HCl, 70 mM sucrose, 1 mM EDTA, and 230 mM mannitol, pH 7.4). The mitochondria-rich pellets were finally collected and stored at −70°C for the individual study.

### Real-time quantitative PCR analysis

Real-time reverse transcription polymerase chain reaction (RT-qPCR) was conducted using LighCycler TaqMan Master (Roche, Germany) in a single capillary tube according to the manufacturer’s guidelines for individual component concentrations. Forward and reverse primers were each designed in a different exon of the target gene sequence, eliminating the possibility for amplifying genomic DNA.

Total RNA was extracted using spin column-based RNA extraction kit (RNeasy Fibrous Tissue Mini Kit, Qiagen) according to protocols provided by manufacturer. Reverse transcriptions were performed with the Transcriptor First Strand cDNA Synthesis Kit (Roche). Briefly, 1 μg of total RNA was mixed with 50 pmole oligo dT and then incubated at 65°C for 10 minutes. After incubation on ice for 5 minutes, 4 μL of 5x reverse transcriptase reaction buffer, 0.5 μL of RNase inhibitor (40 U/μl), 2 μL of dNTP (10 mM for each), and 0.5 μL of reverse transcriptase (20 U/μL) were added into tubes containing hybridized RNA-oligo dT mixtures. RT reactions were carried at 55°C for 30 minutes. PCRs were performed on a Light Cycler (Roche Molecular Biochemicals). Each reaction was carried out with 1 μL of cDNA, 5 μL of 2x reaction mixtures (Maxima Probe qPCR Master Mix), 0.15 μL or each primer (20 μM), 0.2 μL of probe, and 3.5 μL of sterile distilled water. Reactions were performed by incubating in 95°C for 10 minutes, following with 45 cycles of 95°C for 10 sec, 60°C for 30 sec, and 72°C for 1 sec. Analysis of melting curves and determination of threshold cycle (Ct) were performed by the Light Cycler instrument’s software provided by Roche.

### Immunofluorescent (IF) and immunohistochemical (IHC) studies

For IF staining, cryo-sections were fixed with cold acetone for 3 min and then incubated with primary antibody specifically against CD31 (1:200, Serotec) at 4°C overnight. After wash with PBS, sections were incubated Alexa Fluor 594-conjugated goat anti-mouse IgG secondary antibodies for 30 min at room temperature and followed by counter-staining with DAPI. Fluorescent signals were observed with fluorescent-equipped microscope (IX-41, Olympus).

For IHC staining, rehydrated paraffin sections were firstly treated with 3% H_2_O_2_ for 30 min and incubated with Immuno-Block reagent (BioSB) for 30 min at room temperature. Sections were then incubated with incubated with primary antibodies specifically against von Willebrand factor (vWF) (1:200, Millipore), at 4°C overnight. Irrelevant antibodies [(p53, 1: 500 (Abcam) and mouse control IgG (Abcam)] were used as controls in the current study. After washing with PBS thrice, the sections were incubated PolyDetector HRP Label (BioSB) for 30 min and washed with PBS thrice again. Finally, section were covered with DAB substrate-chromogen solution (BioSB) for 5 min and followed by counter-staining with hematoxylin. Three sections of muscle specimens were analyzed in each rat.

For quantification, three randomly selected HPFs (x100 for IHC stain and 400x for IF stain respectively) were analyzed in each section. The mean number per HPF for each animal was then determined by summation of all numbers divided by 9.

#### Vessel density in CLI region

IHC staining for determining the number of vessels was performed with primary antibody against α-SMA (1:400) with incubation at room temperature for one hour, followed by washing with PBS thrice. The anti-mouse-HRP conjugated secondary antibody was then added for 10 minutes, followed by washing with PBS thrice. Three ischemia sections were analyzed in each animal. For quantification, three randomly selected high-power fields (HPFs) (100x) were analyzed in each section. The mean number per HPF for each animal was then determined by summation of all numbers divided by 9.

### Statistical analysis

Quantitative data are expressed as means ± SD. Statistical analysis was adequately performed by ANOVA followed by Bonferroni multiple-comparison post hoc test. Statistical analysis was performed using SAS statistical software for Windows version 8.2 (SAS institute, Cary, NC). A probability value <0.05 was considered statistically significant.

## Results

### Combined cilostazol and clopidogrel treatment increases numbers of circulating EPCs in critical limb ischemia

Flow cytometric analysis was performed to investigate the effect of cilostazol and clopidogrel treatment on circulating endothelial progenitor cells (EPCs) at 18 h and on day 21 after the CLI procedure (Figure 1). Double staining for C-kit/CD31+ (Figure 1-A & B) and Sca-1/CD31+ (Figure 1-C to 1-D); and single staining for CXCR4+ (Figure 1-E & F) and CD34+ (Figure 1-G & H) were used.

At 18 h after the CLI procedure, the number of circulating C-kit/CD31+ cells (Figure 1-A) did not differ among the five groups. On day 21 after the procedure, the number of circulating C-kit/CD31+ cells (Figure 1-B) showed no significant difference between the untreated control group and the CLI only group, or between the control group and the two groups that received treatment with either cilostazol and clopidogrel; but, the number of circulating C-kit/CD31+ (Figure 1-B) cells was significantly higher in all the treatment groups (groups 3–5) than in the CLI group that received no treatment (group 2). Among the treatment groups the number of circulating C-kit/CD31+ cells (Figure 1-B) was notably higher in the group that received the combination therapy (group 5).

At 18 h after the CLI procedure, the number of circulating number of Sca-1/CD31+ cells (Figure 1-C) showed no significant difference between the control group, the CLI only group and the two groups receiving either cilostazol and clopidogrel treatment, or among groups 3 to 5, but was significantly higher in the group receiving the combination treatment (group 5) than in the control group or the CLI group (group 1) with no treatment (group 2). By day 21 after the procedure, the circulating level of these biomarkers revealed no difference among groups 1 to 4 or among groups 3 to 5 (Figure 1-D). However, this circulating biomarker level was still much higher in group 5 than in groups 1 and 2.

At 18 hours after the CLI procedure, the number of circulating CXCR4+ cells (Figure 1-E) displayed no difference between groups 1, 2 and 3, or between groups 4 and 5. However, there were significantly more circulating CXCR4+ cells in groups 4 and 5 than in groups 1, 2 and 3. By day 21 after the procedure, there was no significant difference among the five groups (Figure 1-F).

At 18 hours after the CLI procedure, there was no difference in the number of circulating CD34+ cells (Figure 1-G) among the five groups. By day 21 after the procedure (Figure 1-H), there was no difference in the number of circulating CD34+ cells among groups 1 to 4, but there was a significantly higher number in group 5 than in groups 1 to 4. Together these results suggest that combined cilostazol and clopidogrel treatment increases the number of circulating EPCs.

### Combined cilostazol and clopidogrel treatment decreases inflammation and oxidation in critical limb ischemia

**Figure 2:**
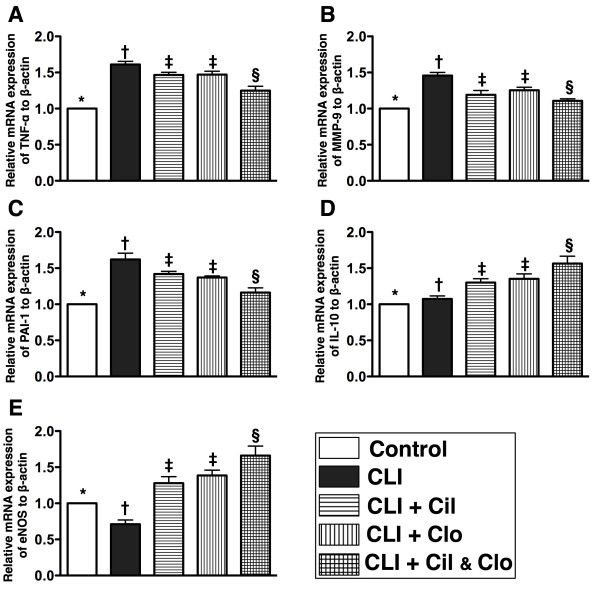
**The mRNA expressions of inflammatory and anti-inflammatory biomarkers in ischemic quadriceps by day 21 after CLI procedure (n = 8). A**, **B** and **C**) indicating mRNA expressions of tumor necrotic factor (TNF-α), matrix metalloproteinase (MMP-9), and plasminogen activator inhibitor (PAI)-1, respectively.* vs. other groups with different symbols, p < 0.0004. **D** &**E**) showing mRNA expressions of interleukin (IL)-10 and endothelial nitric oxide (eNOS), respectively.* vs. other groups with different symbols, p < 0.009. Statistical analysis in each group by ANOVA followed by Bonferroni multiple comparison post hoc test. CLI = critical limb ischemia; Cil = cilostazol; Clo = clopidogrel.

The mRNA expressions of tumor necrotic factor (TNF)-α, matrix metalloproteinase (MMP)-9 and plasminogen activator inhibitor (PAI)-1, three indices of inflammation, were much higher in the untreated CLI group (group 2) than in the control (group 1), and the experimental groups (groups 3, 4 and 5); higher in groups 3 and 4 than in groups 1 and 5, but showed no difference between groups 3 and 4 (Figure 2). Conversely, the mRNA expressions of interleukin (IL)-10 and endothelial nitric oxide synthase (eNOS), two indices of anti-inflammation, were significantly lower in groups 1 and 2 than in groups 3 to 5 and significantly lower in groups 3 and 4 than in group 5, but it revealed no difference between groups 3 and 4. Additionally, eNOS mRNA expression was significantly lower, whereas the IL-10 mRNA expression was higher in group 2 than in group 1.

**Figure 3:**
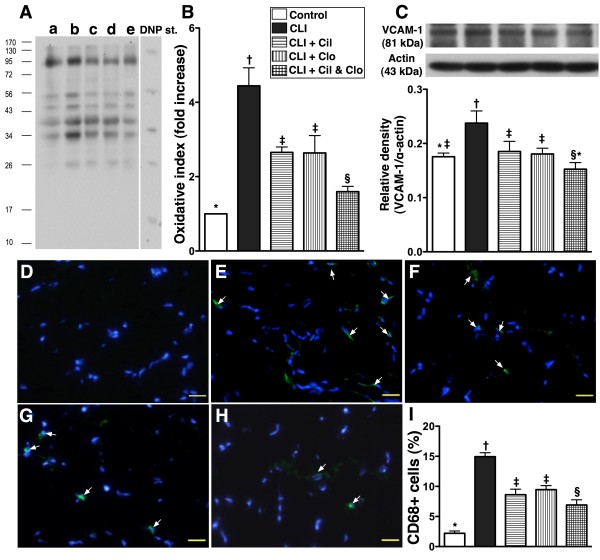
**Western blot result of oxidative stress and protein and cellular inflammatory biomarkers for ischemic quadriceps by day 21 following CLI procedure (n = 8). A**) oxidative index (protein carbonyls) among five groups of animals (a = control, b = CLI only, c = CLI + cilostazol, d = CLI + clopidogrel, e = CLI + combined therapy). **B**)* vs. other groups with different symbols, p < 0.005. (Note: Right lane and left lane shown on upper panel represent control oxidized molecular protein standard and protein molecular weight marker, respectively). DNP = 1-3 dinitrophenylhydrazone. **C**) protein expression of intercellular adhesion molecule (ICAM)-1. **D** to **H**) immunofluorescent (IF) microscopic (400x) findings of CD 68+ cells (white arrows). **I**)* vs. other groups with different symbols, p < 0.0001. Nuclei (blue color) stained with 4’,6-diamidino-2-phenylindole (DAPI) as counter staining. The scale bars in right lower corner represent 20 μm. Statistical analysis in each group by ANOVA followed by Bonferroni multiple comparison post hoc test. CLI = critical limb ischemia; Cil = cilostazol; Clo = clopidogrel.

Oxidative stress (Figure 3-A & B), an indicator of inflammation, was increased in the non-treated CLI group (group 2) in comparison with the other groups, increased in groups 3 and 4 in comparison with group 5, significantly increased in group 5 in comparison with group 1, but there was no difference between groups 3 and 4. Additionally, the protein expression of vascular adhesion molecule (VCMA)-1 (Figure 3-C), another indicator of inflammation, was increased in the untreated CLI group (group 2) in comparison with the other groups, increased in groups 3 and 4 in comparison with group 5, but revealed no difference among groups 1, 3 and 4, or between groups 1 and 5. Furthermore, the number of CD68+ cells, an indicator of inflammatory cell infiltration, as shown by IF staining, showed a similar pattern as oxidative stress among the five groups (Figure 3-D to I).

### Combined cilostazol and clopidogrel treatment reduces skeletal muscle damage in critical limb ischemia

**Figure 4:**
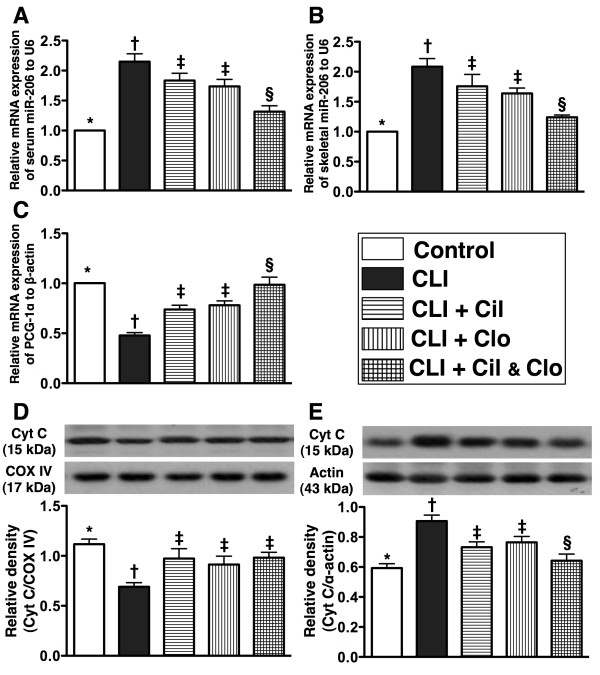
**mRNA and protein expressions of skeletal muscle damage markers in ischemic quadriceps by day 21 following CLI procedure (n = 8). A** and **B**) showing serum and ischemic muscle microRNA-206 levels, respectively. For (A) and (B),* vs. other groups with different symbols, p < 0.0001. **C**) mRNA expression of peroxisome proliferator-activated receptor-γ coactivator-1α (PGC-1α).* vs. other groups with different symbols, p < 0.0001. **D**) mitochondrial cytochrome C (Cyt C) protein expression.* vs. other groups with different symbols, p < 0.008. **E**) Cytosolic cytochrome C protein expression.* vs. other groups with different symbols, p < 0.004. Statistical analysis in each group by ANOVA followed by Bonferroni multiple comparison post hoc test. CLI = critical limb ischemia; Cil = cilostazol; Clo = clopidogrel.

The serum and ischemic muscle microRNA-206 (Figure 4-A and B), an index of skeletal muscle damage, was significantly higher in non-treated CLI group (group 2) than in the other groups, significantly higher in groups 3 and 4 than in groups 1 and 5, significantly higher in group 5 than in group 1, but it showed no difference between groups 3 and 4. Conversely, the mRNA expression of peroxisome proliferator-activated receptor-γ coactivator-1α (PGC-1α) (Figure 4-C), an energy transcriptional coactivator, a major upstream regulator of mitochondrial metabolism and biogenesis, revealed an altered pattern of microRNA-206 among the five groups.

The protein expression of cytochrome C in mitochondria (Figure 4-D), an indicator of energy supply and storage in mitochondria, was significantly lower in the untreated CLI group (group 2) than in other groups, significantly lower in groups 3 to 5 than in the controls (group 1), but similar among groups 3, 4 and 5. In contrast, the protein expression of cytochrome C in the cytosol (Figure 4-E), an indicator of mitochondrial damage, was significantly higher in the untreated CLI group (group 2) than in other groups, significantly higher in groups 3 and 4 than in groups 1 and 5, and significantly higher in group 5 than in group 1, but it exhibited no difference between groups 3 and 4.

### Combined cilostazol and clopidogrel treatment reduces apoptosis in critical limb ischemia

**Figure 5:**
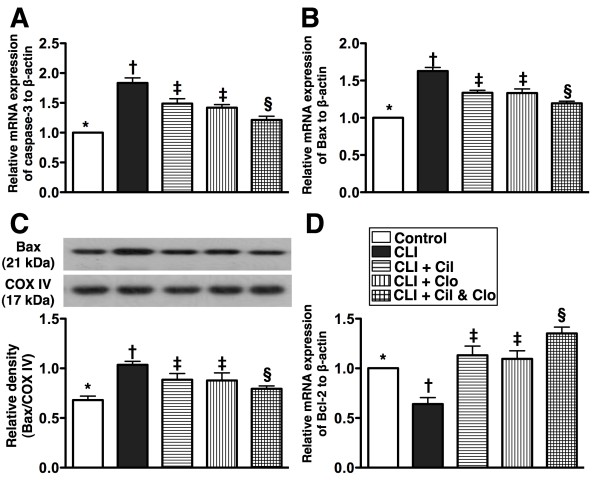
**Protein and mRNA expressions of apoptotic biomarkers in ischemic quadriceps by day 21 following CLI procedure (n = 8). A** and **B**) showing mRNA expression of caspase 3 and Bax, respectively. for (A) and (B), * vs. other groups with different symbols, p < 0.0001. **C**) mitochondrial protein expression of Bax. * vs. other groups with different symbols, p < 0.008. **D**) mRNA expression of Bcl-2. * vs. other groups with different symbols, p < 0.0001. Statistical analysis in each group by ANOVA followed by Bonferroni multiple comparison post hoc test. CLI = critical limb ischemia; Cil = cilostazol; Clo = clopidogrel.

The mRNA expressions of caspase-3 (Figure 5-A) and Bax (Figure 5-B), two indices of apoptosis, were highest in the untreated CLI group (group 2), higher in groups 3 and 4 than in groups 1 and 5, and higher in group 5 than in group 1, but there was no difference between groups 3 and 4. Additionally, the mitochondrial protein expression of Bax (Figure 5-C) displayed a similar pattern as the mRNA expression of Bax among the five groups. In contrast, mRNA expression of Bcl-2 (Figure 5-D), an index of anti-apoptosis, displayed an altered characters among the five groups.

**Figure 6:**
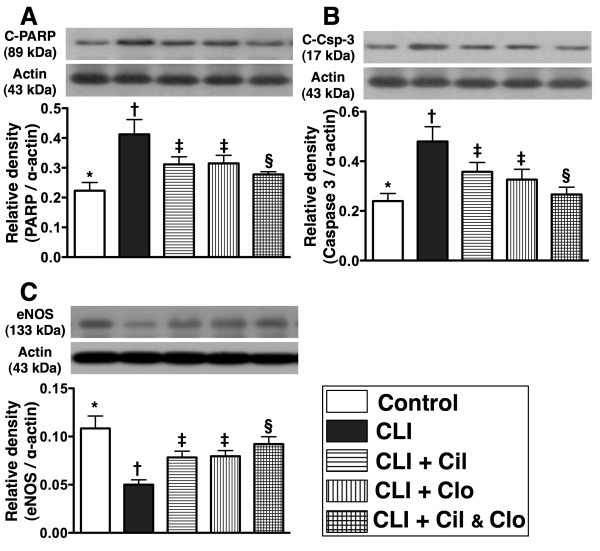
**Western blot results of apoptotic and angiogenic factors in ischemic quadriceps by day 21 following CLI procedure (n = 8). A**) protein expression of cleaved (C) poly(ADP-ribose) polymerase (PARP).* vs. other groups with different symbols, p < 0.006. **B**) protein expression of cleaved casapse 3 (C-Csp-3).* vs. other groups with different symbols, p < 0.004. **C**) protein expression of endothelial nitric oxide synthase (eNOS).* vs. other groups with different symbols, p < 0.002. Statistical analysis in each group by ANOVA followed by Bonferroni multiple comparison post hoc test. CLI = critical limb ischemia; Cil = cilostazol; Clo = clopidogrel.

Poly(ADP-ribose) polymerase (PARP) cleavage (i.e., active form), an indicator of apoptosis, was significantly higher in group 2 than in the other groups, significantly higher in groups 3 and 4 than in groups 1 and 5, notably higher in group 5 than in group 1, but it showed no difference between groups 3 and 4 (Figure 6-A).

Cleaved caspase 3 (i.e., active form) was significantly higher in groups 2 than in other groups, significantly higher in groups 3 and 4 than in groups 1 and 5, higher in group 5 than in group 1, but it showed no difference between groups 3 and 4 (Figure 6-B). These results suggest that less apoptosis occurs in critical limb ischemia after combined cilostazol and clopidogrel treatment.

### Combined cilostazol and clopidogrel treatment increases angiogenesis in the CLI region

**Figure 7:**
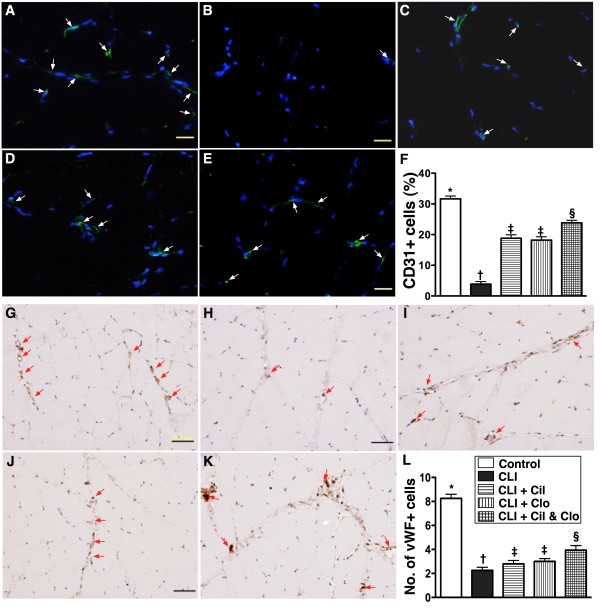
**Quantification of immunofluorescent (IF) (400x) and immunohistochemical (IHC) (200x) staining for ischemic quadriceps by day 21 following CLI procedure (n = 8). A** to **E**) showing the IF stain of CD31+ cells (white arrows) in five groups (A = control, B = CLI only, C = CLI + cilostazol, D = CLI + clopidogrel, E = CLI + combined therapy). **F**)* vs. other groups with different symbols, p < 0.0001. Nuclei (blue color) stained with 4’,6-diamidino-2-phenylindole (DAPI) as counter staining. The scale bars in right lower corner represent 20 μm. **G** to **K**) indicating the results of IHC stain of von Willebrand factor (vWF) + cells (red arrows) in five groups (G = control, H = CLI only, I = CLI + cilostazol, J = CLI + clopidogrel, K = CLI + combined therapy). **L**)* vs. other groups with different symbols, p < 0.0001. The scale bars in right lower corner represent 50 μm. CLI = critical limb ischemia; Cil = cilostazol; Clo = clopidogrel.

**Figure 8:**
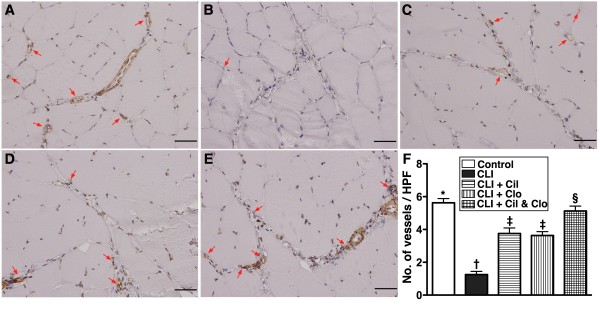
**Immunohistochemical (IHC) (200X) staining of alpha-smooth muscle actin for quantification of small vessel (≤ 15.0 μm) in ischemic quadriceps at day 21 after CLI induction (n = 8). A** to **E**) indicating IHC stain of number of small vessels (≤ 15 μm) (red arrows) in five groups (A = control, B = CLI only, C = CLI + cilostazol, D = CLI + clopidogrel, E = CLI + combined therapy). **F**) * vs. other groups with different symbols, p < 0.001. Statistical analysis by ANOVA followed by Bonferroni multiple comparison post hoc test. Scale bars in right lower corner represent 50 μm. HPF = high-power field (200x). CLI = critical limb ischemia; Cil = cilostazol; Clo = clopidogrel.

The protein expression of endothelial nitric oxide synthase (eNOS) (Figure 6-C), an index of angiogenesis and an anti-inflammatory factor, was lower in the untreated CLI group (group 2) than in the other groups, lower in the two groups that received a single treatment than in the controls (group 1) and the combined treatment group (group 5), significantly lower in the combined treatment group (group 5) than in the controls (group 1), but it did not differ between the two single treatment groups. Additionally, IF staining showed that the numbers of CD31+ and vWF + cells (Figure 7), two indices of angiogenic cells, were significantly lower in the untreated CLI group (group 2) than in the other groups, significantly lower in groups 3 and 4 than in groups 1 and 5, and significantly lower in group 5 than in group 1, but it showed no difference between groups 3 and 4. Furthermore, IHC staining showed that the small number of vessels (<15 μm) in the CLI region exhibited an identical pattern of IF staining among the five groups (Figure 8).

### Blood flow in the CLI region is improved with combined cilostazol and clopidogrel treatment

**Figure 9:**
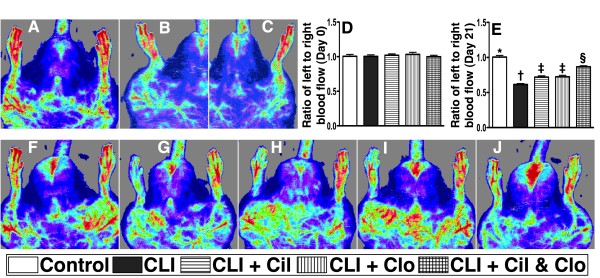
**Laser Doppler scanning of hind limb blood flow on day 21 after CLI induction (n = 8).** (**A**) (simultaneous scanning of both hind limbs), (**B**) (isolated left hind limb scanning) and (**C**) (isolated right hind limb scanning) in normal rats. **D**) Normal blood flow in both hind limbs among five groups prior to the procedure. **F** to **J**) ratio of ischemic/normal blood flow (INBF) among five groups (F = control, G = CLI only, H = CLI + cilostazol, I = CLI + clopidogrel, J = CLI + combined therapy) by day 21 after CLI procedure. **E**)* vs. other groups with different symbols, p < 0.001. Statistical analysis by ANOVA followed by Bonferroni multiple comparison post hoc test. CLI = critical limb ischemia; Cil = cilostazol; Clo = clopidogrel.

The ratio of ischemic/normal blood flow (INBF) did not show any difference among the five groups prior to the CLI procedure. But by day 21 after CLI induction, the INBF ration was significantly reduced in the CLI group receiving no treatment in comparison with the other groups, and was significantly lower in the two groups receiving a single treatment in comparison with the control group (groups 1) and the group receiving cilostazol and clopidogrel combination treatment (group 5), and significantly lower in combination treatment group than in the normal controls, but it displayed no difference between the two groups receiving a single treatment (groups 3 and 4) (Figure 9).

## Discussion

This study investigated the therapeutic effect of combined cilostazol and clopidogrel treatment on a murine model of CLI. Several observations were made: First, CLI without treatment markedly enhanced inflammation, oxidative stress, cellular apoptosis and tissue fibrosis in mice. Second, serum microRNA-206 level was a simple and useful biomarker for determining the skeletal muscle ischemia/damage. Third, cilostazol or clopidogrel therapy used alone showed similar effects on inflammation, oxidative stress, cellular apoptosis, ischemia-related muscle damage and tissue fibrosis, but combination treatment of these two drugs had a more pronounced suppressive effect than either agent used alone. Cilostazol and clopidogrel combination treatment was superior to either drug alone for enriching angiogenic factors and blood vessel density and improving blood flow in the ischemic area.

Inflammation and oxidative stress are two key factors thought to be associated with endothelial dysfunction, and consequent atherosclerosis, plaque rupture and acute arterial occlusive syndrome [17-20]. They are also increased [21-25] and play crucial roles in continued progressive damage to organs after acute ischemic attack [22,25]. One important finding of the current study was that compared to the normal control, inflammation and oxidative-stress were substantially increased in rats after CLI, findings which support previous studies [21-25]. Inflammation and oxidative-stress were significantly suppressed after cilostazol or clopidogrel therapy. A previous study showed that cilostazol therapy markedly suppressed the expressions of the TNF-α and MMP-9, two indicators of inflammation, in lung parenchyma of pulmonary arterial hypertension rats [26]. Additionally, our previous studies and those of others have shown that clopidogrel therapy significantly attenuated circulating levels of soluble CD40 ligand and high-sensitivity C-reactive protein (hs-CRP), two indicators of inflammation, and reduced oxidative stress [27-29]. However, the present study showed that combined cilostazol and clopidogrel therapy more markedly inhibited the inflammatory and oxidative-stress responses, thus, extending the findings of previous studies [26-29].

The association between ischemia and inflammation, and incremental cellular apoptosis and death is well known [24,25,29,30]. In the current study, compared with the normal control, both mRNA and protein levels of apoptosis biomarkers were substantially increased in CLI animals; however, these apoptotic parameters were remarkably reduced in CLI animals after receiving cilostazol or clopidogrel therapy. Conversely, the anti-apoptotic biomarkers which were reduced in CLI animals were markedly upregulated in those CLI animals with cilostazol or clopidogrel therapy. Previous studies have also revealed the effect of cilostazol and clopidogrel therapy significantly inhibited the cellular apoptosis in ischemic tissues/organs [26,29]. Combined therapy with these two agents further ameliorated apoptosis and enhanced the anti-apoptotic effect to a much greater extent than in either agent alone after CLI. Our findings further show that serum and ischemic muscle of levels of microRNA-206, an index of skeletal muscle damage, were further reduced; and mitochondrial cytochrome C and PGC-1α were more significantly preserved with combined cilostazol or clopidogrel therapy in comparison with either treatment used alone.

One particularly distinctive finding in the present study was that the circulating level of EPCs, the protein expression of eNOS, the number of small blood vessels and the numbers of CD31+ and vWF + cells (i.e., new endothelial cells) in CLI region were prominently increased in CLI animals with cilostazol or clopidogrel therapy. Combined treatment with cilostazol and clopidogrel was superior to either treatment used alone for enhancing exhibition of these angiogenic factors both in circulation and in the ischemic region. These findings may explain why the Laser Doppler results of blood flow in CLI area was significantly improved in animals that underwent monotherapy and further improved in animals receiving combined therapy in comparison to those animals without treatment. We have previously demonstrated that cilostazol therapy enhanced the eNOS mRNA expressions in both lung and right ventricle in a rat model of pulmonary arterial hypertension [26]. Additionally, cilostazol has been previously shown to enrich vasculogenesis through enhancing EPC mobilization from bone marrow to circulation and promoting the production of angiogenic factors and chemokines [i.e., eNOS, CXCR4, vascular endothelial growth factor (VEGF) and stromal cell derived factor (SDF)-1α] [30] for recruiting and mobilizing new EPCs/endothelial cells to ischemic region [31,32]. The findings from these studies [26,31,32] have been reinforced by the results from another previous study [33] which has demonstrated that vardenafil, a PDE-5 inhibitor, enhanced ischemia-induced angiogenesis with mobilization of EPCs through a protein kinase G–dependent hypoxia-inducible factor (HIF)-1α/VEGF. Furthermore, it has previously been shown that clopidogrel therapy significantly increased the number of EPCs [34,35]. Moreover, clopidogrel treatment in patients with coronary artery disease not only inhibits platelet activation but also improves endothelial function and nitric oxide bioavailability [36]. Besides, study [36] has further displayed clopidogrel improved adenylyl cyclase-mediated signaling including increased Akt- and eNOS-phosphorylation contributing to improved NO-mediated vasorelaxation [36] and EPC mobilization[34,35]. Accordingly, in addition to reinforcing these previous findings [26,31-36], our results raise the possibility of using a combined regimen for CLI patients who are not candidates for bypass surgery and catheter-based intervention.

### Study limitations

This study has several limitations. First, this study did not investigate the contribution of cilostazol or clopidogrel to anti-thrombosis and anti-platelet activity. Therefore, it is unclear whether reducing thrombosis and platelet activity would potentially contribute to increase in blood flow in the CLI area. Second, the underlying mechanism responsible for the upregulation of EPCs in circulation and the new endothelial cells in CLI region has not been explored in the current study. A previous study suggested that these changes could be due to mobilization from the bone marrow, or alternatively a decrease in the peripheral apoptotic destruction of EPCs [34]. Third, the results of the current study did not address whether the mechanisms of cilostazol and clopidogrel therapy were the same or different and if the high dosage of cilostazol or clopidogrel might work as well as both of them in the moderate for increasing EPC number in circulation and generating eNOS and angiogenesis in ischemia area.

**Figure 10:**
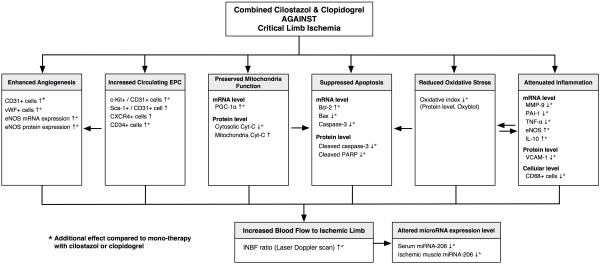
**Proposed mechanisms underlying the effects of different treatment on CLI in a rat model based on the findings of the present study.** EPC = endothelial progenitor cell; eNOS = endothelial nitric oxide synthase; vWF = von Willebrand factor; PGC-1α = peroxisome proliferator-activated receptor-γ coactivator-1α; Cyt C = cytochrome C; PARP = poly(ADP-ribose) polymerase; MMP-9 = matrix metalloproteinase; PAI-1 = plasminogen activator inhibitor 1; TNF-α = tumor necrotic factor-α; IL-10 = interleukin-10; VCAM-1 = vascular cell adhesion molecule 1; miRNA = microRNA; INBF = ratio of ischemic to normal blood flow.

In conclusion, our results demonstrated that cilostazol and clopidogrel combined therapy has a synergic effect on rat CLI. The proposed mechanisms are summarized in Figure 10. This finding may expand the potential applications of this therapeutic modulation not only for CLI patients but also for patients with intracranial arterial obstructive syndrome [37].

Kun-Chen Lin indicates equal contribution in this study compared with the first author.

Jenq-Lin Yang indicates equal contribution in this study compared with the correspondence author.

## Competing interests

The authors declare that they have no competing interests.

## Authors’ contributions

SJJ, LKC, TCY, HKY and SL designed the experiment, drafted and performed animal experiments. TTH, SS, YCH, SCK, CHW and CYL were responsible for the laboratory assay and troubleshooting. YJL, CHWand HKY participated in refinement of experiment protocol and coordination and helped in drafting the manuscript. All authors report no disclosures and have any commercial associations or interests, including consultancies, stock ownership or other competing equity interest. All authors have read and approved the final manuscript.
